# Effectiveness of an e-health system on depression among patients with somatic disorders

**DOI:** 10.1192/j.eurpsy.2023.988

**Published:** 2023-07-19

**Authors:** T. Vitcheva, G. Hadlaczky, N. G. Petros, V. Carli

**Affiliations:** National Centre for Suicide Research and Prevention of Mental Ill-Health, Karolinska Institutet, Stockholm, Sweden

## Abstract

**Introduction:**

Patients with severe somatic conditions frequently develop depressive symptoms, with a reduction in quality of life, sleep disturbances and suicide as some of the most serious consequences. However, there is a lack of evidence-based interventions to reduce this comorbidity. The NEVERMIND system aims to address this issue by collecting biomedical and psychometric data through a smart shirt and questionnaires, which are used to predict patients’ depressive symptoms. Based on the predictions, patients are offered personalised feedback to self-manage their mental health symptoms in the form of lifestyle behavioural advice, mindfulness-based therapy, and cognitive behavioural therapy.

**Objectives:**

The primary objective was to assess the effectiveness of the NEVERMIND system in reducing depressive symptoms in patients with somatic conditions in comparison to treatment as usual. Some of the secondary objectives were to examine the NEVERMIND system’s effectiveness in preventing new depressive symptoms, sustaining the effects of the intervention at 24 weeks post-baseline, and reducing suicide ideation.

**Methods:**

For this pragmatic randomised controlled trial, 425 patients diagnosed with myocardial infarction, breast or prostate cancer, kidney failure, or lower limb amputation were recruited from hospitals in Turin, Pisa and Lisbon. Data from clinical interviews and structured questionnaires was collected at baseline, 12 weeks, and 24 weeks. The primary outcome was depressive symptoms at week 12 as measured by the Beck Depression Inventory II (BDI-II), while secondary outcomes included prevention of depressive symptoms, suicide ideation, self-reported general interest, satisfaction with daily life, illness perception, self-compassion, and the sustainability of the system’s effect at 24 weeks post-baseline. The intention-to-treat analyses included all patients, while the per-protocol analyses included 333 patients.

**Results:**

The intervention group included 213 and the control group 212 patients, with the sample’s mean age being 59.41 (SD=10.70). Those who used the system had statistically significant lower depressive symptoms at 12 weeks (mean difference=-3.05, p=0.004; 95%CI -5.12 to -0.99) compared to controls, with a clinically relevant effect size (Cohen’s *d*=0.41). Notable significant reductions included suicide ideation (mean difference =-0.61, p=0.020; 95%CI -1.13 to -0.10) and incidence of depressive symptoms at week 12 (OR=0.43, p=0.019; 95%CI 0.22 to 0.87). The improvements in depressive symptoms were sustained at week 24 (mean difference =-1.34, p=0.015; 95%CI -2.41 to -0.26). No significant differences were observed for other secondary outcomes.

**Conclusions:**

The NEVERMIND system was shown to be superior to standard care in reducing and preventing depressive symptoms among the studied sample.

**Disclosure of Interest:**

None Declared

